# Exosomal Micro-RNAs as Intercellular Communicators in Idiopathic Pulmonary Fibrosis

**DOI:** 10.3390/ijms231911047

**Published:** 2022-09-20

**Authors:** María Cristina Negrete-García, Javier de Jesús Ramos-Abundis, Noé Alvarado-Vasquez, Eduardo Montes-Martínez, Martha Montaño, Carlos Ramos, Bettina Sommer

**Affiliations:** 1 Molecular Biology Laboratory, Department of Research in Pulmonary Fibrosis, National Institute of Respiratory Diseases “Ismael Cosío Villegas”, Calzada de Tlalpan 4502, Col. Sección XVI, Mexico City 14080, Mexico; 2 Higher School of Medicine Instituto Politécnico Nacional, Salvador Díaz Mirón esquina Plan de San Luis S/N, Miguel Hidalgo, Casco de Santo Tomás, Mexico City 11340, Mexico; 3 Biochemistry Department, National Institute of Respiratory Diseases “Ismael Cosío Villegas”, Calzada de Tlalpan 4502, Col. Sección XVI, Mexico City 14080, Mexico; 4 Cell Biology Laboratory, Department of Research in Pulmonary Fibrosis, National Institute of Respiratory Diseases “Ismael Cosío Villegas”, Calzada de Tlalpan 4502, Col. Sección XVI, Mexico City 14080, Mexico; 5 Bronchial Hyperreactivity Research Department, National Institute of Respiratory Diseases “Ismael Cosío Villegas” Calzada de Tlalpan 4502, Col. Sección XVI, Mexico City 14080, Mexico

**Keywords:** exosomes, extracellular vesicles, miRNA, exosomal miRNAs, idiopathic pulmonary fibrosis

## Abstract

Communication between neighboring or distant cells is made through a complex network that includes extracellular vesicles (EVs). Exosomes, which are a subgroup of EVs, are released from most cell types and have been found in biological fluids such as urine, plasma, and airway secretions like bronchoalveolar lavage (BAL), nasal lavage, saliva, and sputum. Mainly, the cargo exosomes are enriched with mRNAs and microRNAs (miRNAs), which can be transferred to a recipient cell consequently modifying and redirecting its biological function. The effects of miRNAs derive from their role as gene expression regulators by repressing or degrading their target mRNAs. Nowadays, various types of research are focused on evaluating the potential of exosomal miRNAs as biomarkers for the prognosis and diagnosis of different pathologies. Nevertheless, there are few reports on their role in the pathophysiology of idiopathic pulmonary fibrosis (IPF), a chronic lung disease characterized by progressive lung scarring with no cure. In this review, we focus on the role and effect of exosomal miRNAs as intercellular communicators in the onset and progression of IPF, as well as discussing their potential utility as therapeutic agents for the treatment of this disease.

## 1. Introduction

The pulmonary microenvironment is constituted of heterogeneous cell groups with different functions, and understandably, an adequate communication between them is indispensable in maintaining homeostasis and physiological processes [1,2]. Not long ago, chemokines, cytokines, growth factors, and adhesion molecules were considered the principal protagonists in intercellular communication. But recently, extracellular vesicles (EVs) have gained importance due to their substantial role as intercellular communicators via the transfer of their cargo to neighboring or distant cells [3,4,5,6]. EVs are structures delimited by a lipid bilayer of diverse sizes, shapes, and distinct biogenesis pathways. According to their size, EVs have been classified into exosomes (30–120 nm), microvesicles (MVs, 50–1000 nm), and apoptotic bodies of 50–2000 nm in diameter [3,6,7]. Exosomes play important roles in cell-to-cell communication, tissue repair, immune response and organism development [8,9]. Their content influences many cellular functions such as cell proliferation, differentiation, angiogenesis and modulation of the immune system [5,9,10]. It has been suggested that EVs can act as biomarkers for the diagnosis and prognosis of different respiratory diseases, since they are secreted by different cell types, both in normal cellular processes and pathological conditions [11,12,13]. Their content or cargo is heterogeneous, it is composed of proteins, lipids, and nucleic acids such as microRNAs (miRNAs), and is associated with their origin and the cellular microenvironment [6,14,15]. The cargo can be internalized by the recipient cell mainly by endocytosis or by direct fusion of exosomes with the membrane of the target cell to deliver their content into the cytosol [3,16,17]. The cargo delivery modifies and redirects the biological functions of the recipient cell, partially in response to the miRNAs present, and consequently regulating the post-transcriptional gene expression, differentiation, proliferation, and cell-to-cell interaction by repressing or degrading their target mRNAs [18,19].

On the other hand, idiopathic pulmonary fibrosis (IPF) is a progressive chronic interstitial lung disease of unknown etiology characterized by scar tissue accumulation and the histological picture of usual interstitial pneumonia (UIP), leading to a progressive decline of lung function with generally an average survival of 3–5 years after diagnosis, poor prognosis, with no cure and, consequently with few therapeutic options [20,21]. The incidence and prevalence of IPF increases with age and is diagnosed mostly in male patients older than 65 years. In this context, the immune system’s role in developing IPF has been widely discussed. Although many of the innate immune cells participate in mediating the inflammatory process, the role that they may play in the long run has been questioned [22], a reason why the importance of inflammation in the IPF etiology is still controversial and sometimes considered an epiphenomenon of fibrosis. Some evidence reported an increase in absolute values of neutrophils, macrophages, eosinophils, and epithelial cells in induced sputum from IPF patients in comparison to healthy subjects [23]. Moreover, an increase in the expression of defensins (DEFA3 and DEFA4) in acute exacerbations of IPF versus controlled IPF was detected [24]. The polarization of macrophages has also been involved with IPF. Although the M2 phenotype is normally considered anti-inflammatory, when the injury is persistent this phenotype is responsible for secreting pro-fibrotic factors such as TGFβ, PDGF, and VEGF which induces the activation and transformation of fibroblasts [25].

Similarly, some reports suggest that the adaptative immune system also plays an active role in IPF. In IPF patients, circulating B cells were more antigen differentiated, with greater plasmablast proportions, and interestingly, the extent of this differentiation correlated with IPF patient lung volumes [26]. An imbalance in the Th1/Th2 population of T lymphocytes has been proposed, suggesting that an increase in the Th2 phenotype, associated with the profibrotic cytokines, may be involved in the progression of the condition. This is also supported by the antifibrotic properties of IFN γ, related to the Th1 subset [27]. 

Based on all this evidence that supports an important role of immune response in IPF development, the use of immunomodulators for its treatment was seen as an option to consider. However, there are no effective immunosuppressive therapies. Some results show that resistance to corticosteroids mediated by the glucocorticoid receptor β might develop [28,29]. The Th1/Th2 theory seemed to be also ineffective, since the inhibition of IL-13, an important Th1 mediator, had no effect on the outcome of IPF patients [30]. Even though immunotherapy is regarded as non-effective, there is evidence that suggests potential of some modern anti-fibrotic therapies which may be involved in the immune system response. An example is the application of IFN γ alongside pirfenidone to normal human lung fibroblasts stimulated with TGFβ1 and PDGF, which had a synergic effect in attenuating fibroblast proliferation, migration, and differentiation [31]. There is also an interesting effect using the combination of pirfenidone and nintedanib; these drugs showed a potential to diminish the secretion of cytokines by activated B cells and induced a decrease in the migration and activation of fibroblasts treated with a conditioned medium of activated B cells [32]. 

In the beginning, IPF was considered a chronic inflammatory disorder with the development of progressive fibrosis. However, recent evidence indicates that it is a consequence of an epithelial-driven disorder which is associated with environmental and genetic risk factors, aging-associated processes, and profibrotic epigenetic reprogramming [33]. Although pathogenic features of IPF include bronchiolization of the distal airspace and the presence of atypical airway cell types associated with loss of terminal bronchioles in regions of fibrosis, the role of the airway epithelium in the pathogenesis of IPF is unidentified. However, some recent results showed that healthy and IPF airway epithelia are biophysically distinct and were regulated by pathologic activation of the ERBB-YAP axis, which shows one of the probable mechanisms regulating airway epithelial-driven fibrosis [34]. Likewise, results obtained from surgically resected bronchi and peripheral lung tissues in both 31 IPF patients and 39 control subjects, showed that the areas of mucus glands (MUC5B+) were meaningfully bigger in IPF patients in comparison to control subjects. In addition, in the epithelium from bronchi, and proximal and distal bronchioles a higher MUC5B and MUC5AC expression by secretory cells, as well as a minor number of ciliated cells, linked with an increased ciliary length were observed in IPF patients, which suggest that mucus hypersecretion and ciliary impairment in conducting airway are involved with the alveolar injuries in IPF patients [35]. And recently, an over-expression of 23 genes associated with epithelial dysfunction, with probable activation of different pathways related to immune response, and apoptosis was reported. These results give new knowledge and suggest that targeting these pathways and mainly those related to the secreto-protein/mucin dysfunction could be helpful in the treatment of IPF [36].

In recent years, the importance of exosomes in IPF and their contribution to disease pathogenesis, have been gaining relevance. For example, one of the first studies performed with EVs from bronchoalveolar lavage fluid (BALF) of IPF patients, showed that these EVs function as carriers of WNT5a signaling mediator and thus contribute to disease pathogenesis [37]. It was also demonstrated that the antifibrotic effect of activated fibroblasts is carried out by exosomes that contain antifibrotic prostaglandins (PGE_2_) [38]. Nevertheless, studies that evaluate the role and effects of miRNAs exosome content (exosomal-miRNAs) on the recipient cell are still scarce. Therefore, this review will discuss the recent reports describing the effects of exosomal-miRNAs as intercellular communicators and their effects on IPF progression. 

## 2. Exosomes

### 2.1. History and Discovery

The term exosome was used for the first time to describe the microvesicles (MVs) with 5ʹnucleotidase activity and secreted by neoplastic cell lines [39]. In 1983, Harding and Johnstone, discovered that during the maturation of blood reticulocytes, small vesicles associated with transferrin receptors were released, into the extracellular space through endocytosis and recycling [40]. During reticulocyte differentiation and with ultrastructural studies, it was evidenced that the vesicles released from multi-vesicular bodies (MVBs) fused with the plasma membrane, which were later named exosomes [41,42,43]. In 1989, Peters et al. showed that during the interaction of the T lymphocytes with their target cells, there was a release of numerous membrane vesicles contained in the cytolytic granules [44]. A decade later, Raposo et al. demonstrated that the exosomes released from Epstein-Barr virus (EBV)-transformed B lymphocytes were implicated in both antigen presentation and activation of T-cells [45]. Until then, the functions of exosomes were not fully elucidated. In 2007, Valadi et al. reported a novel mechanism of genetic exchange between cells that was mediated through mRNA and miRNAs molecules contained within the exosomes [46].

### 2.2. Composition of Exosomes

The quantities, content, and membrane composition of exosomes are variable and depend on their origin and cellular status [47,48]. In general, EVs are made up of cytoskeletal, cytosolic, heat shock, plasma membrane proteins, and proteins involved in vesicle trafficking [19]. EVs express several types of proteins on their surface, for instance T and B cell receptors, cytokines and cytokine receptors, integrins, and lectins [49]. Besides, the membrane composition of EVs is highly dynamic, heterogeneous and dependent on the cellular source and environmental conditions [50]. EVs are released by almost all cell types such as platelets, tumor cells, dendritic cells, T and B lymphocytes, eosinophils, epithelial cells, endothelial cells, and mesenchymal stem cells (MSCs) [51]. Currently, some databases on EV composition as EVpedia, Exocarta and Vesiclepedia [52,53,54] include the most recent information about their composition. It is well established that EVs-cargo depends on stimuli that they receive under different physiological or pathological conditions [55]. Among the main biomolecules are proteins, lipids, and nucleic acids (DNA, mRNA, miRNA, lncRNA). However, sorting mechanisms that control the specific RNA cargo in exosomes are still not well understood. As already mentioned, EVs can disrupt signaling networks in neighboring and distant cells through intercellular delivery of their functional cargo (e.g., nucleic acids, proteins, lipids) [19]. For this reason, EVs have been associated with neuronal communication [56], antigen presentation [10,57] and immune response [58,59] as well as with pathological processes such as cancer progression [60,61], cardiovascular diseases [62,63], inflammatory processes [12,64], neurodegenerative ailments [65,66] and respiratory diseases such as asthma, chronic obstructive pulmonary disease (COPD), and idiopathic pulmonary fibrosis (IPF) [51,67,68].

### 2.3. Biogenesis of Exosomes

EVs are classified into three types according to their biogenesis mechanisms. Exosomes originated from the endocytic pathway. MVs, which bud from the plasma membrane, and apoptotic bodies that are released through blebbing and cell membrane fragmentation of apoptotic cells [55,69]. However, the overlapping of physical characteristics, as well as the difficulty to isolate each class of EVs, led the International Society for Extracellular Vesicles to recommend the use the generic term “EV” as appropriate, instead of a specific term for each class of EV [70]. Accordingly, in this review we indistinctly use the terms EVs and exosomes. 

Exosomes originate from the endosomal system through three different stages [6]: (1) Formation of endocytic vesicles by invagination of the plasma membrane and their release inside the endosomes as intra-luminal vesicles (ILVs) [71,72,73]; (2) Development of multivesicular bodies (MVBs) that originates by inward budding of the endosomal membrane [74]; and (3) The degradation of MVB or its fusion with the plasma membrane to release the ILVs as exosomes [3,75]. 

**Figure 1:**
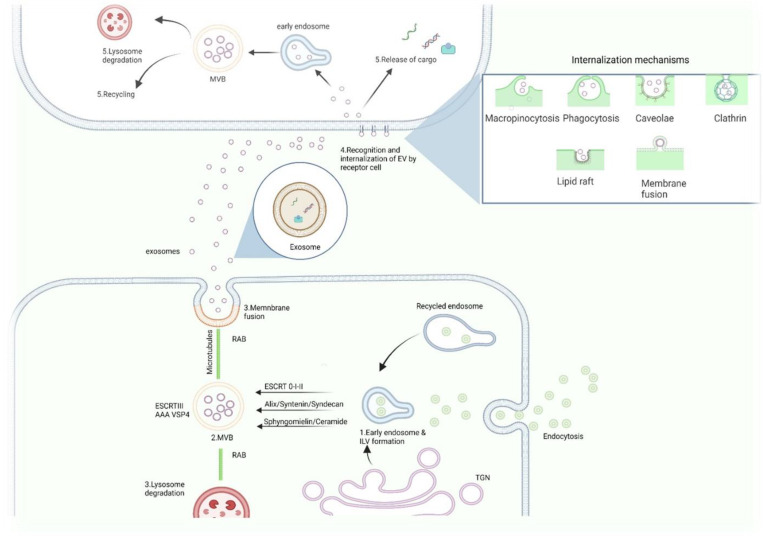
Biogenesis and Exosomes-Uptake. 1. Formation of early endosomes from the TGN and EVs-uptake. 2. Formation of MVB through ESCRT-dependent and independent methods. 3. Different pathways of ILV inside of the parent cell: degradation by lysosome or secretion to extracellular space. 4. Recognition of EVs by receptor cell and internalization through different means. 5. Fate of internalized EVs: release of cargo into a recipient cell to induce effect, degradation through the lysosome pathway, or recycling to repeat the cycle. EVs: extracellular vesicles; ILV: intraluminal vesicles; MVB: multivesicular body; RAB: ras related in brain GTPase; TGN: trans golgi network.

The endosomal sorting complex required for transport (ESCRT) machinery drives the formation of MVBs and ILVs, which are composed of approximately thirty proteins. These proteins assemble in four complexes: ESCRT-0, -I, -II, and -III associated in turn with accessory proteins (VPS4, VTA1, and ALG-2, and interacting protein X (Alix)) [6,47,74,76]. In general, MVB/ILV formation pathways are divided into two types: (1) The ESCRT-complex dependent pathway, and (2) The ESCRT-complex independent. In the ESCRT-dependent pathway, the machinery recognizes ubiquitylated proteins, and in the ESCRT-complex independent recognizes sphingomyelinases, sphingosine-1-phosphate, and tetraspanin-enriched domains [77]. In the ESCRT-independent pathway, the formation of MVBs/ILVs is not entirely dependent on the ESCRT complex [9]. In this sense, in 2009, Stuffers et al. demonstrated that in spite of the depletion of key subunits of all ESCRTs complexes, it was still possible to observe the formation of MVBs using electron and confocal microscopy [78]. This process includes neutral sphingomyelinase (nSMase) which induces membrane curvature, invagination, and exosome formation through ceramide and tetraspanins that act in sorting exosome cargo (Figure 1). Both ESCRT-dependent and ESCRT-independent mechanisms for MVB biogenesis occur in mammalian cells [69,79,80]. On the other hand, the intercellular transport of exosomes is mediated by their binding to cytoskeleton proteins like dynein and myosin and some GTPases [81], while the EVs fuse with the plasma membrane through the soluble N-ethylmaleimide-sensitive factor attachment protein receptor (SNARE) complex [82,83].

### 2.4. Exosomes–Uptake

Intercellular communication between neighboring or distant cells can be through the release and uptake of exosomes in recipient cells. It has been established that the content of the exosomes is dependent on the stimuli that the progenitor cells have received previously [19], and that its content or cargo act as important molecular messengers in different biological and pathological processes [84]. Besides, differences in the size and/or surface components of exosomes can influence their recognition and internalization by the recipient cells [19,85]. Exosome-uptake and cargo delivery into the extracellular medium of the acceptor cell occurs in three steps, however, the specific mechanism is still under study. The first step involves the recognition of the EV by the acceptor cell, although the selection parameters are unknown [86,87]. The second step is the internalization of EVs by the recipient cell through a variety of pathways including clathrin-dependent endocytosis (micropinocytosis and phagocytosis), caveolin-mediated uptake, lipid raft-mediated internalization, or by direct fusion [17,80,88] (Figure 1). Other participating proteins localized on the surface of EVs and/or acceptor cells are the tetraspanins, integrins [3], lipids, lectins, heparan sulfate proteoglycans, and extracellular matrix (ECM) components [50,54]. For instance, the ECM can act as a “zipper” between the integrins of exosomes and the recipient cell [80]. To date, it has not been possible to identify a specific receptor for exosome internalization. Exosome uptake might depend on the acceptor cell type rather than on the vesicles themselves [87]. The third and last step is when the exosomes are internalized and then maybe recycled, re-secreted, or selected for degradation by the lysosome pathway [89,90]. An additional alternative suggested, is exosome fusion with the plasma membrane of the recipient cell, and the posterior release of its cargo directly into the cytosol [3]. Notwithstanding, it is to be highlighted that it is unknown whether the exosome uptake mechanisms are dependent on the localization, degradation, and/or function of the different components of the exosome’s cargo [50].

## 3. miRNA Biogenesis

miRNAs are classified as short non-coding RNAs with a length of approximately 17–25 base pairs in their mature forms [91], and whose primary function is the post-transcriptional regulation of gene expression [92]. The first description of these short RNAs was made in 1993 by Lee et al. who reported the presence of miRNAs in *Caenorhabditis elegans* [93]. Currently, there are 38,589 entries on miRbase v. 22.1 of which 1917 are human [94]. miRNA biogenesis starts with the transcription of non-coding sequences and intronic parts of protein-coding genes by RNA polymerase II to create a pri-miRNA with a cap structure and a poly A tail [95,96]. Subsequently, this pri-miRNA is processed by a Microprocessor complex, which is constituted by a RNasa III (Drosha), a double-stranded RNA binding protein DiGeorge critical region 8 (DGCR8) and cofactors that transform the pri-miRNA into pre-miRNA [97,98] that is exported from the nucleus into the cytoplasm via Exportin 5 in a GTP dependent process [99]. At the cytoplasm, it is processed by the endonuclease Dicer into a mature miRNA duplex conformed by a passenger strand and a guide strand [100]. Then, the loading of the dsRNA into the RNA-induced silencing complexes (RISC) and Argonaute protein (AGO2) select the guide strand and pairs the “seed region” to target mainly the 3′UTR of mRNAs, while the passenger strand is degraded [101,102].

## 4. miRNAs Sorting into Exosomes

The specific mechanisms that control the miRNAs sorting into exosomes remain unknown. A study reported the existence of sequence motifs present in miRNAs, and the sumoylation through proteins like hnRNPA2B1, which control their localization into exosomes. In this same study, microarray analysis demonstrated that upon cellular activation, the profile of miRNAs in the cells is different from their exosomes, which suggests that the sorting process is highly specialized [103]. It was also, described that post-transcriptional modifications such as 3´end adenylation and uridylation contribute to the degree of enrichment of miRNAs into exosomes [104]. McKenzie et al. showed that the lack of AGO2 protein was related to a decrement in the miRNA content in exosomes [105], while Shurtleff et al. reported that RNA binding protein Y box protein 1 (YBX1) is required to sort miRNAs into exosomes [106]. Of interest too, is the neutral sphingomyelinase 2, which is important both for exosome biogenesis and as a regulator of exosomal miRNA secretion [84].

## 5. Exosomal-miRNAs as Intercellular Communicators

Inter-cellular communication includes signaling molecules and/or direct contact between cells [107]. In this complex landscape, non-coding RNAs like miRNAs have an important role, especially by the significant effects observed after their delivery to target cells [108]. The first evidence of the horizontal transfer of genetic information through EVs and the effects on the recipient cell were described in 2006. In this work, the transfer of exosomal mRNA and proteins from embryonic stem cells to hematopoietic progenitor cells was demonstrated [109]. In 2007 Valadi et al. provided the first evidence of the exosomal mRNA transfer between mouse donor cells and human recipient cells in an in vivo assay [46]. Shortly after, in 2008 Al-Nedawi, K et al., reported that oncogenic activity could be transferred through MVs and they named such vesicles “oncosomes” [60]. All these works indicated that the transfer of RNAs and proteins from exosomes, confers new functional and biological properties to the recipient cell. 

Based on these findings, the protein and miRNAs enclosed into exosomes sparked interest as possible biomarkers. Taylor, D et al. demonstrated that exosomal miRNA profiling of circulating exosomes from tumors could be used as diagnostic marker between patients with benign ovarian disease and ovarian cancer [110]. Despite this evidence, the utility of exosomal miRNAs as probable prognostic biomarkers in tumoral diseases, and proofs of functional miRNA transfer with neighboring and/or distant recipient cells were scarce. In 2010 Pegtel M et al. hypothesized that miRNAs transferred through exosomes might have an important function as intercellular communicators by inhibiting the expression of their mRNA targets. To confirm this hypothesis, the authors used as a model, EBV infection, demonstrating that miRNAs from exosomes of EBV-infected cells were transferred and delivered to subcellular sites repressing the gene expression in uninfected recipient cells [111]. In the same context, two years later the transfer of exosomal miRNAs between DCs was reported, confirming a new cell model of posttranscriptional regulation through exosomes [18]. A year later, Ismail et al. demonstrated that EVs-miRNAs derived from macrophages induce differentiation of their target cells, a fact that supports the role of EVs-miRNAs in the development of immune functions [112]. Considering all this information, the importance of EVs as vehicles of intercellular communication is confirmed as a relevant issue for future work.

## 6. Exosome Functions in Respiratory Pathologies

It is well known that in degenerative chronic pulmonary diseases, a response is generated by the resident progenitor cell populations to promote the regeneration and repair of tissue damage [113,114]. This process requires cell-intrinsic factors and the interaction of all the cells present in the lung microenvironment [115]. In this context, exosomes act as paracrine mediators through the transfer of their biological cargo, which influences the repair, remodeling and regeneration of the lung. Thus, they have a potential role in lung regenerative medicine [70,87,116]. Because exosomes are present in various biological fluids (e.g., urine, plasma) [117,118], and in fluids from the respiratory tract such as BAL [119], nasal lavage, saliva, and sputum [120,121,122], exosomal-miRNAs might be useful as biomarkers for both diagnostic and prognosis in multiple lung diseases including IPF [51,68,123]. Worth mentioning is the exciting advances in the therapeutic use of exosomal-miRNAs derived from MSCs, mainly in chronic degenerative lung disease such as pulmonary fibrosis, COPD, asthma, and pulmonary arterial hypertension (PAH) recently reported [124,125,126,127].

## 7. Idiopathic Pulmonary Fibrosis (IPF)

IPF is a chronic progressive disease that affects the lung interstitium, changing it and decreasing the lung´s ability to carry out its function [128]. Its exact origin is unknown, but a plethora of risk factors has been identified. These include ageing, environmental factors such as smoking, certain comorbidities, and certain specific genetic mutations involved with the maintenance of telomeres and proteins like mucin [129]. IPF is a disease with higher prevalence and incidence in patients over 65 years in the USA [130]. Its median survival is approximately 3 years after diagnosis [131]. Although there is no concrete mechanism by which this disease originates, some evidence points to the role of repetitive micro injuries at the level of the alveolar epithelium, with special emphasis on alveolar epithelial cells type II (AECII) [33]. This continuous stress releases a myriad of pro-fibrotic factors such as TGFβ, PDGF, TNF, and osteopontin to name some examples [129]. The release of these molecules induces cellular senescence, activation and proliferation of fibroblasts and their transformation into myofibroblasts, a cell type deeply involved with IPF pathology, and with the decrease in pulmonary function [20,132]. Some of the most studied pathways implicated in the development of fibrosis are TGFβ and WNT pathways, that are also involved with the production of ECM generation of myofibroblasts, and regulation of cell senescence [133].

## 8. Exosomes in IPF

**Figure 2:**
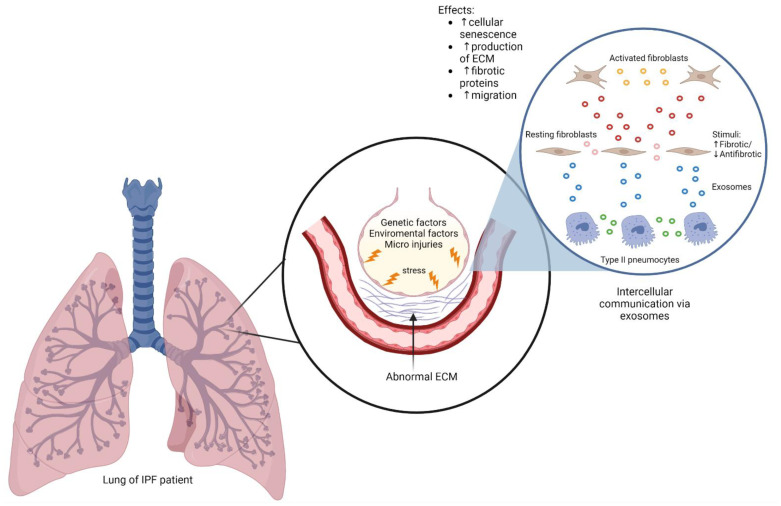
Abnormal cellular communication during IPF. The combination of risk factors and direct injuries on the alveolar epithelium causes an increase in stress. Type II pneumocytes are involved in repair mechanisms, however, aberrant stimuli induce the synthesis of pro-fibrotic factors and through intercellular communication mediated by EVs that activate fibroblasts promoting the increased production of abnormal ECM and loss of functional tissue on the parenchyma.

Isolated studies have shown that intercellular communication mediated by exosomes is essential in the activation of pro-fibrotic pathways in IPF [134,135] (Figure 2). Likewise, some evidence points out an increase in the exosome synthesis in patients with IPF [37,136]. These studies prompted an increased number of recent works about the functionality and effect of exosomes in respiratory diseases such as IPF. A condition prevalent in IPF is a hypoxic microenvironment, which per se promotes an increment in the exosome production through over-expression of RAB protein, a key participant during the release process of exosomes [137]. Exosomes derived from AEC of IPF patients and cultured in a hypoxic microenvironment (hypoxic exosomes), showed an increase in the long no-coding RNA (lncRNA) HOTAIRM1, which in turn, promoted lung fibrosis through the miR-30dp3/YY1/HSF1 axis [134]. In 2018 Martin-Medina et al. found an increment in the number of EVs present in BALF of IPF patients and in mice challenged with bleomycin (BLM) in comparison with healthy controls. Moreover, the same authors reported an increase in WNT5a, a protein associated with the proliferation and activation of fibroblasts in EVs derived from primary human fibrotic lung fibroblasts (HFLF), and from fibroblasts activated with TGFβ [37]. Another study showed that EVs isolated from the serum of IPF patients had over-expression of CD19, CD69, CD8, and CD86 on their membrane, which relates exosomes with the immune response [138].

On the other hand, EV-miRNAs have also been detected in different respiratory fluids in IPF, like BALF and sputum, and have been evaluated for their probable role as potential biomarkers [139]. In 2018, Liu B et al. were the first to report the downregulation of exosomal-miR-30a in BALF from IPF patients. Through a quantitative analysis, they revealed that miR-125b, miR-128, miR-21, miR-100, miR-140-3p, and miR-374b-p were upregulated by more than two-fold in patients with IPF in comparison with healthy subjects. In contrast, let-7d, miR-103, miR-26 and miR-30a-5p were downregulated. A dual luciferase reporter assay confirmed a regulatory association between miR-30a-5p and its target gene TAB3. An overexpression of miR-30a-5p decreased TAB3, α-SMA and FNT expression in A549 cells stimulated or not with TGF-β treatment. Therefore, a decreased expression of this miRNA in the BALF of patients with IPF may be linked with the TAB3 increased expression, so it could be an important factor in IPF progression [140]. 

In induced sputum samples of IPF patients, the presence of three exosomal-miRNAs as probable biomarkers for diagnosis of this ailment (miR-142-3p, miR-33a-5p and let-7d-5p) was reported. After studying their diagnostic value, the authors suggested that the three miRNAs signature could be useful for IPF detection and diagnosis associated with the severity of the disease [141]. In the same context, Kaur et al. recently compared the miRNA profiles of BALF and lung tissue-derived exosomes of healthy non-smokers, smokers, and patients with COPD or IPF. Authors identified three differentially expressed exosomal-miRNAs in the BALF, and only one in the lung-derived exosomes from COPD patients compared to healthy non-smokers. Of these, miR-122-5p was down-regulated in COPD patients in comparison to healthy non-smokers and smokers. Also, there were 55 differentially expressed exosomal-miRNAs derived from lung tissues of IPF patients compared to non-smoking controls. After analyzing their results, the authors did not detect a unique miRNA signature that could serve as a potential biomarker to identify the disease progression of these chronic pulmonary diseases [68]. In their work, Lacedonia et al. analyzed only five exosomal miRNAs derived from the serum of IPF patients in comparison with healthy controls [142]. These miRNAs (miR-16, miR-21, miR-26a, miR-210, and let-7d), had previously already been involved in the IPF pathogenesis, with the exception of miR-16 which had been linked only with hepatic fibrosis [143]. Interestingly, the authors found that the five mentioned miRNAs were down-regulated, including mir-26a and let7d which have antifibrotic functions [144], and miR-21 and miR-210 that have profibrotic roles [145]. The authors concluded that more studies were necessary to confirm their results [142]. The proteomic analysis of EVs-cargo derived from fibroblast cell lines LL97A and LL29 isolated from lungs of IPF patients was recently published, and the results were compared to those derived from the fibroblast cell lines CCD8Lu and CCD19Lu isolated from healthy donors. A total of 86 differentially expressed proteins were identified in each comparison group. These results revealed proteins involved in fibrogenic processes, such as TNC, IGFBP7, FBN1, COL1A2, COL1A1, LOXL1 in Evs cargo isolated from IPF cell lines. And after KEGG enrichment analysis pathway, the authors pointed out that all those proteins participated in focal adhesion, PI3K-Akt, and ECM–receptor interaction signaling pathways involved in IPF pathogenesis [146].

## 9. Exosomal-miRNAs as Intercellular Communicators in IPF

Until recently, the impact of exosomal-miRNA in airway diseases, especially in IPF, had been not sufficiently emphasized. This was probably a consequence of a controversial paper published by Cheville et al., who suggested that the copy number of miRNAs in each EV was very low and possibly had no biological effect, and this assumption created a certain skepticism about EV´s functional role in vivo. The authors reported that regardless of the exosome source (plasma, seminal fluid, mast cells, or ovarian cancer cells), over 100 exosomes were necessary to observe one copy of a given miRNA, which would significantly limit its effect on the cell or organism [147]. However, some recent articles reported using optimized methods for exosome purification, confirming both in vitro and in vivo many different biological effects derived from the isolated exosomes [7,75,148,149].

The role of exosomes in IPF is a current study issue, and the increasing number of publications about this topic further supports its relevance. Therefore, we made a revision and analysis of the most recent papers that evaluated both the importance of exosomal- miRNAs as intercellular communicators, and as protagonists in the onset and progression of IPF (Table 1).

As far as we know, the first study reporting the presence of exosomal miRNAs in serum of IPF patients was made by Makiguchi et al. These authors reported the presence of serum exosomal miR-21-5p in a bleomycin fibrosis mouse model (BLM-fibrosis mouse model) as well as in serum from IPF patients. They suggest that the overexpression of miR-21-5p could be clinically associated with the risk of death in IPF [13].

The loss of Thy-1 (CD90) expression had been previously shown to correlate with active fibrogenesis in IPF due to its role as a regulator of myofibroblast differentiation. Moreover, Thy-1-integrinβ5 heterotypic interaction (in trans) also contributes by inhibiting the myofibroblastic differentiation induced by TGFβ [150,151]. In 2017 Shentu et al. investigated if Thy-1 expression was important in fibroblast uptake of EVs from MSCs (m-EVs) in comparison with EVs derived from normal human lung fibroblasts (NHLF) (f-EVs). Additionally, the authors evaluated their role in myofibroblast differentiation and reported that m-EVs, but not f-EVs, suppressed TGFβ-induced myofibroblastic differentiation in a Thy-1 (CD90) dependent way, and moreover, that the interaction of Thy-1-β-integrins facilitated the mEVs uptake by fibroblasts. They also demonstrated the presence of miR-630 in m-EVs, but not in f-EVs, and that it plays an anti-fibrotic role during the differentiation of myofibroblasts by inhibiting the expression of different profibrotic genes [152]. In the study performed by Yao et al. it was reported that lung tissue of a BLM-fibrosis rat model had an over-expression of miR-328 and a under-expression of the family with sequence similarity 13-member A (FAM13A) gene. Authors examined the role of exosomes derived from alveolar M2 Macrophages (AM2Mfs) of the BLM-fibrotic rat model and co-cultured with pulmonary interstitial fibroblasts. The results showed an over-expression of COL1A1, COL3A1, and ACTA2 (α-SMA) genes as well as a promotion in the proliferation of the fibroblasts. Finally, in in vivo studies where exosomal miR-328 was silenced using an antagomir, the fibrotic lung area was reported to be significantly inhibited, which was related to a decrease in the number of α-SMA and collagen-I positive cells. Results support the importance of inhibiting the expression of exosomal miR-328 and the regulation of its target gene FAM13 expression to attenuate the development of PF [153]. In addition to the role of EVs as intercellular communicators, the importance of some transmembrane proteins as regulators of the specific content within EVs has been recently reported. An example is Syndecan-1 protein, which besides participating in the exosome’s biogenesis, intervenes as a regulator in miRNA sorting into exosomes in lung tumorigenesis [154]. Syndecan-1 controls lung epithelial migration and adhesion processes [155]. A recent study performed in 2019 by Parimon et al. demonstrated that Syndecan-1 is overexpressed by AECII in IPF patients and in the BLM-fibrosis mouse model. Functional assays made in Syndecan-1 wild-type (WT) and with Syndecan-1 deficient (Sdc1^−/−^) mice treated with bleomycin, showed that Syndecan-1 promotes the proliferation and expansion of fibroblasts; moreover, it induces the epithelial reprogramming to the fibrotic phenotype through signaling pathways that involve TGFβ and Wnt/β-catenin. When EVs isolated from BALF of fibrotic lungs (F-EVs) were re-instilled intratracheally into the BLM-fibrosis-mouse model (WT and Sdc1^−/−^), they showed that F-EVs exacerbated lung fibrosis in the WT-BLM-mouse model in comparison with Sdc1^−/−^ animals. Similar results were obtained in co-culture assays using lung epithelial cells with F-EVs from mice that were WT and with Sdc1^−/−^ BLM-fibrosis. Therefore, the authors concluded that F-EVs in fibrotic lungs increased the fibroproliferative signals through TGFβ and Wnt/β-catenin signaling pathways activity, and that Syndecan-1 facilitates the effect of F-EVs by reprogramming lung epithelial cells to the profibrotic phenotype. With additional experiments using miRNA-Seq of F-EVs isolated from the different BLM-mouse models, authors observed that F-EVs from WT mice, in comparison with Sdc1^−/−^ mice, had significant under-expression of the anti-fibrotic miR-503-5p, miR-34-b-5p, miR-144-3p, miR-142-3p. Additionally, they found that miR-144-3p and miR-142-3p had a similar trend of decreased EV levels in IPF patients versus control subjects, with an analogy to WT versus Sdc1^−/−^ animal models, respectively. Thereby, they concluded that Syndecan-1 controls the packaging of antifibrotic miRNAs into EVs. Finally, they found that mice treated with Sdc1^−/−^ EVs had reduced lung fibrosis compared with those receiving WT-EVs. The collagen content was also significantly reduced in Sdc1^−/−^ EV-treated mice, while WT fibrotic mice did not show incremental fibrosis compared to saline controls. All these experiments demonstrated that Syndecan-1drives lung fibrosis in vivo through the regulation of EV-cargo [156].

Another study that supports the importance of exosomes as cell communication vesicles was carried out in 2020 by Kadota et al. The authors evaluated whether EVs isolated from conditioned media (CM) of lung fibroblasts obtained from IPF subjects (F-EVs) or of EVs of NHLF (NF-EVs), could transfer their miRNAs to human bronchial epithelial cells (HBECs) and induce IPF-related phenotypic alterations. In the coculture of F-EVs with HBECs, a p21 and p16 over-expression were observed, and notably a positive staining of β-galactosidase, suggesting a probable role of F-EVs in cell senescence induction. In addition, an increase both in the intracellular levels of ROS (inROS) and in mitochondrial ROS (mtROS) production were found, a fact that could be associated with the aberrant activation of the DNA damage response (DDR). Authors also compared the profiles of miRNAs-cargo between F-EVs and NF-EVs and found six miRNAs significantly upregulated (miR-19a-3p, miR-23b-3p, miR-127-3p, miR-145-5p, miR-424-5p, miR-494-3p) in the F-EVs. Mitochondrial damage and the presence of senescence characteristics in the epithelial cells were associated with the transfer of miR-23b-3p and miR-494-3p to HEBC and with the inhibition of their specific targets SIRT3 [135].

It is well known that the cell-free secretome from stem cells can elicit protection and higher regeneration than the cells alone [157]. In 2020 Dinh and et al. evaluated the effect of lung spheroid cell’s-secretome (LSC-Sec), or lung spheroid cell exosomes (LSC-Exo) on lung regeneration in fibrosis mouse models induced by silica or bleomycin. Previous evidence showed that LSC-Sec and mesenchymal stem cell secretomes (MSC-Sec) attenuated fibrosis in mouse models, BLM-fibrosis models and silica-fibrosis mouse models, concluding that both treatments reduced fibrosis by preserving alveolar epithelial structures. Considering that a secretome is not only comprised of soluble proteins but also exosomes, the authors evaluated whether tissue regeneration could be attributed to the exosomal miRNAs found in LSC-exosomes, and compared the results with those observed using MSC-exosomes of a BLM-fibrosis rat model. In both cases protective effects that maintain normal lung architecture and attenuate the fibrotic process, lung apoptosis, and collagen deposition were observed. The differential expression profiles of miRNAs showed that among the 42-upregulated miRNAs in LSC-Exo, miR-99a-5p and miR-100-5p and the anti-fibrotic miR-30a-3p were significant, while let-7a-5p and let-7f-5p were the most upregulated in the MSC-Exo. However, the potential targets of these expressed miRNAs were not determined, a question that should be answered in the following research reports. Additional experiments showed that both LSC-Sec as well as LSC-Exo promote lung repair in pulmonary fibrosis [158]. 

It has been recognized that bone mesenchymal stem cells (BMSCs) have the property of repairing injured tissues [159,160,161,162]. In this regard, in 2020 Wan et al. determined that the EVs derived from BMSCs inhibit proliferation, migration, invasion, and differentiation of the HFLF cell line (LL29). Likewise, a lower expression of miR-29 in lung tissues of IPF patients compared with tissues from healthy subjects was observed, confirming its anti-fibrotic role through regulation of genes such as COL1A1 and COL3A1. By exploring the mechanism by which BMSCs-EVs inhibited HFLF activation and IPF progression, HFLF cells were transfected with a miR-29 mimic, and as a result the inhibition of fibroblasts differentiation into myofibroblasts was observed. When the BMSC-EVs were transfected with a miR-29b-3p inhibitor and co-cultured with the HFLFs, their proliferation, migration, and invasion capacity were increased. Similarly, BMSC-EVs suppressed IPF progression in the IPF mouse model. Additional experiments showed that miR-29b-3p from BMSCs-EVs directly targets FZD6. Therefore, the authors concluded that the protective effect of miR-29b-3p obtained from BMSCs-EVs happens through downregulation of its target FZD6, which may provide a novel treatment for IPF [163].

In the same year, another study evaluated whether the miRNAs derived from exosomes from sera of a BLM-fibrosis mouse model were involved in the fibroblast-to-myofibroblast differentiation in IPF. To verify this, the differential expression profiles of miRNAs extracted from exosomes of sera in both mice treated or not with bleomycin was determined. miR-22 was upregulated and its role in myofibroblast differentiation was studied. The transfection of human embryonic lung fibroblasts (HELF) with miR-22 mimic, or with miR-22 inhibitor in cells stimulated or not with TGFβ1, showed that miR-22 mimic transfection induces a decrease in α-SMA expression. In contrast, an over-expression of this gene was reported in the presence of miR-22 inhibitor. Additionally, miR-22 decreased the phosphorylation of ERK1/2 and the expression of connective tissue growth factor (CTGF) induced by TGFβ1. Finally, when miR-22 mimic was administrated after bleomycin challenge in the fibrosis mouse model, the collagen content in the lungs and the α-SMA expression were attenuated too, suggesting that exosomal miR-22 could be a therapeutic agent for the treatment of IPF [164].

Meanwhile, Guiot et al. assessed the impact of exosomal miRNAs on the progression of IPF, focusing specifically on the activity of miR-142-3p, which was significantly upregulated in exosomes from sputum and plasma of patients with IPF. The authors had previously observed a positive correlation between the levels of exosomal miR-142-3p with the percentage of sputum macrophages in IPF patients. Therefore, they decided to evaluate the biological effect of this miRNA in AECs (A549 cell line) and in lung fibroblasts (LF) (MRC5 cell line) transfected with miR-142-3p mimics. Results showed a reduction both in the expression of TGFβRI mRNA, as well as in cellular proliferation. Therefore, they concluded that miR-142-3p had anti-fibrotic properties. Additionally, they studied the effect of exosomes obtained from macrophages on the expression of profibrotic genes. In co-culture assays from AECs and LF and THP1 macrophage-exosomes, they observed an increment of miR-142-3p levels in both cell lines, demonstrating that these exosomes can transfer miR-142-3p to the recipient cells. These vesicles were able to suppress profibrotic activation both in epithelial cells and in lung fibroblasts [165].

Another recent work evaluated the effect of miRNA-EVs on the physiology and pathogenic process of IPF by studying EVs from human bronchial epithelial cells (HBEC- EVs), and EVs from human small airway epithelial cells (HSAEC-EVs). In normal human primary lung fibroblasts (NHPLF) stimulated with TGFβ, and co-cultured with each EV type, it was observed that HBEC-EVs attenuated TGFβ-induced myofibroblasts differentiation by suppressing the expression of COL1A1and ACTA2 more efficiently than HSAEC-EVs. When the effect of these EVs as promoters of senescence on lung epithelial cells was examined, it was found that HBEC-EVs inhibited p21 expression and β-galactosidase induced by TGFβ. Additional experiments showed that both canonical and non-canonical WNT signaling pathways were the main mechanisms for HBEC-EVs mediated suppression of myofibroblast TGFβ-induced differentiation. It was also reported that 25 of the 30 miRNAs highly expressed in HBEC-EVs were downregulated in IPF lung samples, whereas 5 miRNAs were upregulated. The bioinformatic analysis of these miRNAs, determined that the 30 miRNAs present in HBEC-EVs negatively regulate TGFβ signaling, with a concomitant effect on WNT pathways. The analysis of the RNA-seq data of the recipient cells (NHPLF), reflected upstream participation of WNT5A, WNT3A, WNT1 and WNT10B, although only WNT5A and WNT10B were detectable by qRT-PCR. Among the 16 miRNAs targeting WNT5A were miR-26a, miR-26b, miR-141a, and miR-200a, which are included in the 30 most abundant in HEBC-EVs; while among the 19 miRNAs targeting WNT10B, were miR-16, miR-29, miR-29c and miR-148a. In mimic transfection assays of these miRNAs, it was demonstrated that only transfections with miR-16 and miR-148a mimics significantly suppressed WNT10B expression. And moreover, the anti-fibrotic properties of miR-16, miR-26a, miR26b, miR-141, miR148a, and miR-200a were confirmed by their ability to suppress TGFβ-induced myofibroblast differentiation. Additional experiments suggested also the likelihood that this specific miRNAs-cargo was responsible for HBEC senescence via regulation of the WNT signaling pathway. After probing the anti-fibrotic properties of HBEC-EVs in a BLM-mouse model, the results showed a significant attenuation of BLM-induced lung fibrosis, a consequence of diminution in the β-catenin expression in the lungs of these EV-treated mice. Likewise, the senescence markers p16 and p21 were clearly suppressed by HBEC-EVs treatment via negative regulation of TGFβ-WNT crosstalk. Taking these results together, the HBEC-EVs can be a promising cell-free antifibrotic modality for the treatment of IPF, via TGFβ-WNT signal pathways crosstalk [166].

In 2021 Inomata et al. analyzed the exosomal-miRNAs profile of serum from a BLM-fibrosis mouse model and in animals without challenge. They found over-expression of exosomal-miR-16 on day 14, in comparison to animals without bleomycin treatment, and decided to study the role of miR-16 in fibrosis both in vivo and in vitro assays. Interestingly, an anti-fibrotic effect in a BLM-fibrosis mouse model treated with miR-16 mimic administration on day 14 was observed. In these animals, the secretion of secreted protein acidic and rich in cysteine (SPARC) in serum, a protein involved with ECM formation and activated by the mTORC pathway, was inhibited. Additionally, miR-16 mimic or a negative control oligo was transfected into normal lung fibroblasts (HFL-1). Results obtained showed that miR-16 mimic significantly inhibited rapamycin-insensitive companion of mTOR (Rictor) expression in these lung fibroblasts. Therefore, the antifibrotic role of miR-16 in lung fibrosis by inhibiting the mTORC2-SPARC axis was demonstrated [167,168].

On the other hand, Zhou et al. investigated whether the release of miR-186, an anti-fibrotic miRNA in IPF, and its presence in the BMSC-EVs could interfere in the progression of IPF in a murine model. They co-cultured HFLF and BMSC-EVs and observed that the viability and invasiveness of the lung fibroblasts were significantly diminished, while apoptosis showed a significant increase after coculture with BMSC-EVs. By Western-blot and RT-qPCR analysis they identified a reduced expression of α-SMA and collagen I in this fibroblasts culture. When the effect of BMSC-EVs in a BLM-fibrosis mouse model was studied and compared with untreated animals, the results showed a decrease in collagen synthesis and a reduction in myofibroblastic markers. As already mentioned, miR-186 plays an antifibrotic role in IPF, an observation confirmed by the down-expression of this miRNA in lung tissues of IPF patients in comparison to control subjects. The highest expression levels of miR-186 were observed in the BMSC-EVs from control subjects, compared to BMSCs alone. Moreover, the effect of this specific miRNA released from BMSC-EVs was investigated both in vitro and in vivo assays. To study the effect on fibroblasts, transfection assays with miR-186 inhibitor, and negative control (NC) inhibitor into BMSCs were performed. The results showed that the expression of miR-186 was diminished in HLFF co-cultured with BMSC-EVs transfected with miR-186 inhibitor, compared to the HLFF treated with BMSC-EVs and with NC inhibitor. In contrast, the proliferation, migration and invasion were significantly incremented and a significantly higher expression of α-SMA and collagen I in these same fibroblasts was detected. Bioinformatic analysis pointed out SRY-related HMG box transcription factor 4 (SOX 4) as a key transcription factor involved in the progression of IPF. Therefore, they investigated whether miR-186 affected IPF by targeting SOX4 and its downstream gene, Dickkopf-1 (DKK1). When the authors investigated the effect of treatment with BMSC-EVs on PF in the BLM-fibrosis mouse model, they observed a decreased expression of α-SMA and collagen 1, which was increased after miR-186 expression in BMSCs. Moreover, they demonstrated that EV-miR186 could target SOX4 and downregulate DKK1 to alleviate the occurrence of IPF [169,170,171].

Although the therapeutic utility of EVs derived from umbilical cord-derived MSCs (uMSCs-EVs) and BMSC-EVs had been reported in previous studies [162,172,173], the molecular mechanisms involved are still only partly understood. In this regard, Shi et al. studied the effect of uMSCs-EVs in comparison with MSCs alone in a BLM-fibrosis mouse model. Results showed that both treatments improved the survival rate and body weight of BLM-challenged mice in comparison to mice treated with PBS. In addition, the degree of lung tissue damage and collagen deposition were also improved. Likewise, a reduction in the expression levels of α-SMA, fibronectin (FNT), TGFβII and TGFβRII in lung tissues of mouse models treated with either treatment was described. Additional results confirmed that uMSC-EVs prevented myofibroblast differentiation by inhibiting the TGFβ signaling pathway in a normal mouse lung fibroblast (NMLF) cell line incubated with TGFβ. When the levels and functions of miRNAs enriched in uMSC-EVs were evaluated, it was found that miR-21-5p, miR-23a-3p, miR-125b-5p, let-7f/a, and miR-145-5p were highly expressed in the uMSC-EVs. On the other hand, in silico analysis, reported that miR-21-5p and miR-23a-3p directly target TGFβII and TGFβRII, respectively. Additional experiments showed that uMSC-EVs could inhibit myofibroblast differentiation by miR-21-5p and miR-23-3p and the down-expression of their respective targets TGFβ2 and TGFβR2 [174].

Recently, Santos-Álvarez et al. analyzed the differential expression of miRNAs in the EVs-cargo obtained from two lung fibrotic cell lines (LL29 and LL97) and compared them with the results obtained from a normal human lung fibroblasts cell line (CCD19). After bioinformatic analysis, it was shown that 77 miRNAs were upregulated and 68 down-regulated. Moreover, they highlighted the presence of 117 novel miRNAs. After pathway enrichment analyses, potential target genes involved with cell proliferation, regulation of apoptosis, pathways in cancer, and proteoglycans in cancer were defined. Therefore, the authors suggested that miRNAs contained in EVs-cargo could be helpful as biomarkers for fibrogenesis, diagnosis, and therapeutic intervention of IPF [175].

**Table 1:** Exosomal miRNAs and their effects in IPF disease.

Sample “Donor Cell”	EVs Source	Recipient-Cell	MiRNA-Cargo in EVs and Function	Target	Major Biologic Effects	Possible Mechanism Associated	EV Isolation	Perspective in IPF Disease	Ref
**BMSCs NHLF (CCL210)**	BMSCs-CM NHLF-CM	NHLF -TGFβ induced HFLF-TGFβ induced	↑ miR-630 (anti-fibrotic)	N-cadherin	↓ Fibroblast differentiation	↓ profibrotic gene expression α-SMA, Col3a1	UC	Further studies to know the mechanism of action of miRNAs of BMSC-EVs	[152]
**AM2Mfs of BLM-fibrotic rat model**	AM2Mfs -CM (exosomes)	Interstitial fibroblasts	↑ miR-328 (profibrotic)	FAM13	↑ Proliferation	↑ profibrotic gene expression α-SMA, Col1a1, Col3a1	PEG and UC	Exosomal-miR-328 derived of AM2Mfs aggravate PF via FAM13	[153]
**WT-BLM fibrosis mouse model and Sdc1^−/−^ BLM-fibrosis mouse model**	BALF	LEC re-instilled intratracheally	↓ miR-503-5p, ↓ miR-34b-5p, ↓ miR-144-3p and ↓ miR-142-3p (anti-fibrotic)	↑ MUC5b TGFβRI	↑ Fibroblast proliferation	↑ Lung fibrosis by activation of TGFβ and WNT/β catenin signaling pathways	UF	Syndecan-1 induces ↑ profibrotic pathways and controls miRNA-cargo	[156]
**HFLF and NHLF**	HFLF-CM NHLF-CM (exosomes)	HBEC	↑ miR-19a-3p, ↑ miR-23b-3p, ↑ miR-127-3p, ↑ miR-145-5p, ↑ miR-424-5p, ↑ miR-494-3p	↓ SIRT3	mitochondrial damage and senescence in epithelial cells	Exosomal ↑ miR-23b-3p and ↑ miR-494-3p ↑ mtROS in epithelial cells	UC	Accelerated epithelial -cell mitochondrial damage and senescence is caused via-exosomal miRNAs	[135]
**LSC-secretome and MSCs-secretome**	Secretome-Exosomes	BLM-fibrotic rat model and Silica-fibrosis mouse model	↑ miR-99-5p, ↑ 100-5p, ↑ 30a-3p in LSC-Exo and ↑ let-7a-5p, ↑ let-7f-5p in MSC-Exo	ND	ND	ND	UF	LSC-Sec as well LSC-Exo promotes lung repair in pulmonary fibrosis	[158]
**LL29 HFLF, hBMSCs, BLM-fibrotic mouse model**	BMSC-CM-EVs	No transfer assays	↑ miR-29b-3p (anti-fibrotic)	FZD6, αSMA, Collgen I	EVs inhibit fibroblast proliferation, migration, invasion, and differentiation	↓ WNT-β catenin signaling pathway	UC	EVs as possible therapeutic agent	[163]
**BLM-fibrotic mouse model and HELF-TGF** **β**	Serum (Exosomes)	No transfer assays	↑ miR-22	CTGF and alpha SMA	miR-22 inhibits fibroblasts differentiation	Inhibition of ERk1/2 phosphorylation-TGFβ induced	EQ™	miR-22 as probable therapeutic agent	[164]
**Sputum and plasma of IPF patients and healthy subjects**	THP1-CM (Exosomes)	A549 and MRC5	↑ miR-142-3p (anti-fibrotic)	TGFβRI COL1A1 and COL3A1	Reduce the expression of profibrotic genes and TGFβRI	Repression of fibrotic response TGFβ-induced	UC	New therapeutic strategy	[165]
**HBEC BMSCs, BEASB-2B, NHDF, HSAEC**	HBEC-CM BMSC-CM EVs	NHLF	↑ miR-26a, ↑ miR-26b, ↑ miR-141a, ↑ miR-200a and ↑ miR-16, ↑ miR-29, ↑ miR-29c and ↑ miR-148a	Wnt-5a WNT10	Attenuation both myofibroblast differentiation and cellular senescence	Inhibition of TGFβ-WNT signaling pathways	UC	New therapeutic strategy	[166]
**BLM-mouse model**	Serum (Exosomes)	No transfer assays	↑ miR-16 (anti-fibrotic)	SPARC	Attenuation of hydroxy-proline content in the lungs of BLM-treated mice	Inhibition of mTORC pathway via mTORC2/SPARC axis	EQ™	New therapeutic strategy	[167]
**Healthy-BMSCs**	BMSCs-CM EVs	HFLF (LL29 cells) and BLM-fibrosis mouse model	↑ mir-186 (anti-fibrotic)	↓ αSMA ↓ Col1a1 ↓ SOX4 ↓ DKK1	↓ Fibroblast activation, ameliorating of IPF	Inhibition of WNT signaling pathway	UC	New therapeutic strategy	[169]
**human-uMSCs**	uMSCs-CM EVs	NMLF and BLM-fibrosis mouse model	↑ miR-21-5p ↑ miR-23-3p (anti-fibrotic)	↓ TGFβII and ↓ TGFβRII	Alleviate PF by ↑ AEC proliferation and ↓ myofibroblast differentiation	Inhibition of TGFβ signaling pathway	UC	New therapeutic strategy	[174]
**HFLF (LL29 and LL97) HNLF (CCD19)**	HFLF-CM NHLF-CM EVs	No transfer assays	↑ 77 miRNAs and ↓ 68 miRNAs	ND	ND	In vitro approach	UC	In vitro approach	[175]

**AEC** = alveolar epithelial cells; **AM2Mfs** = alveolar M2 macrophages; **BALF** = bronchoalveolar lavage fluid; **BLM** = bleomycin; **BMSCs** = bone marrow stem cells; **CM** = conditioned medium; **EVs** = extracellular vesicles; **EQ** = ExoQuick™ **HBEC** = human bronchial epithelial cell; **HFLF** = human fibrotic lung fibroblasts; **HSAEC** = human small airway epithelial cells; **LSC** = lung spheroid cell; **LEC** = lung epithelial cells; **NMLF** = normal mouse lung fibroblast; **mtROS** = mitochondrial reactive oxygen species; **ND** = not done; **NHDF** = normal human dermal fibroblasts; **NHLF** = normal human lung fibroblasts; **PEG** = polyethylene glycol; **PF** = pulmonary fibrosis; **UC** = ultracentrifugation; **UF** = ultrafiltration; **uMSCs** = umbilical cord-mesenchymal stem cells.

## 10. Discussion and Conclusions

The heterogeneity of cells in the pulmonary microenvironment makes an adequate communication between them indispensable. Central participants in this communication are exosomes, which influence different aspects of the cell or organism. These effects are derived from their cargo, which is constituted of proteins, lipids, and nucleic acids as for instance miRNAs. miRNAs especially, have an important role derived from their capacity to regulate post-transcriptional gene expression by repressing or degrading their target mRNAs. In idiopathic pulmonary fibrosis (IPF), which is a chronic, fatal lung disease with few therapeutic options, recent data suggested the probable advantages of the exosomal-miRNAs for its treatment. Some examples of the latter are mentioned in this review, where the antifibrotic effect of exosomal-miRNAs obtained from bone marrow mesenchymal cells (Exosomal-BMSC) were reported [152,166,169,174]. Although these studies showed interesting results, when used in vivo models that mimic the human IPF, they have several disadvantages. For example, the BLM-fibrosis mouse model is not the better model to evaluate the development of IPF since it is reversible, but notwithstanding, remains the most used. Another option is the silica-fibrosis mouse model, but unfortunately this is also not the best mouse IPF model since it induces more aggressive fibrosis and is irreversible. Other models used in the study of IPF are cell cultures derived from both healthy subjects and IPF patients. This has advantages such as the very detailed and specific information about the cells utilized, including their origin, and their phenotypic and genetic characteristics that facilitate the acquisition of new knowledge about exosomal-miRNAs. Even though the study of exosomes is a topic that is currently being actively worked on, the technical difficulties in isolating exosomes, as well as the absence of a standard protocol for their therapeutic use limit their clinical application. Moreover, its use is not without risks, since miRNAs may have a fibrotic or anti-fibrotic role, which makes it necessary to additionally determine the stimuli that induce the synthesis and cargo of the miRNAs found in the exosomes. Although some miRNAs have shown excellent results to attenuate the fibrotic process in IPF, their clinical application still faces different challenges, such as easy degradation by RNA enzymes, no targeting, and low stability in vivo. Additionally, determining how the great numbers of exosomal-miRNAs interact in the cell is another enormous challenge that must be overcome.

Although this review was focused on the analysis of exosomal-miRNAs in IPF, the most recent data about exosomal-miRNAs in malignant pathologies [176], diabetes mellitus [177], other lung diseases [178], and emergent diseases such as COVID-19 [179], clearly highlight the importance of further advancing this line of research.
